# Association of hospital-diagnosed sleep disorders with suicide: a nationwide cohort study

**DOI:** 10.1192/j.eurpsy.2022.341

**Published:** 2022-09-01

**Authors:** N. Høier, T. Madsen, A. Spira, K. Hawton, M. Benros, M. Nordentoft, A. Erlangsen

**Affiliations:** 1 Mental Health Centre Copenhagen (CORE), Danish Research Institute For Suicide Prevention, Hellerup, Denmark; 2 Mental Health Center Copenhagen, Core-copenhagen Research Center For Mental Health, Hellerup, Denmark; 3 Johns Hopkins Bloomberg School of Public Health, Department Of Mental Health, Baltimore, United States of America; 4 University of Oxford, Centre For Suicide Research, Oxford, United Kingdom; 5 CORE-Copenhagen Research Center for Mental Health, Mental Health Center Copenhagen, Hellerup, Denmark

**Keywords:** Suicide, suicide prevention, Suicidology, sleep disorder

## Abstract

**Introduction:**

Sleep disorders and psychiatric disease are closely related, and psychiatric diseases are associated with elevated suicide risks. Yet, the association between sleep disorders and suicide remains to be assessed using a consistent measure of sleep disorders.

**Objectives:**

The aim of this study was to examine whether people with a hospital-diagnosis of sleep disorders had higher suicide rates than people with no diagnosis.

**Methods:**

In a cohort study, nationwide data on all persons aged 15+ years living in Denmark during 1980-2016 were analysed. Sleep disorders were identified through diagnoses recorded during contacts to somatic hospitals. Incidence Rate Ratios (IRR) were estimated using Poisson regression models and adjusted for relevant covariates.

**Results:**

In all, 3,674,563 males and 3,688,164 females were included, of whom 82,223 (2.2%, mean age: 50.2, SD: 17.5) males and 40,003 (1.1%, mean age: 50.6, SD: 19.9) females had sleep disorder diagnoses. Compared to those with no sleep disorders, the adjusted IRRs for suicide were 1.6 (95% CI, 1.4-1.7) and 2.2 (95% CI, 1.8-2.6) for males and females with sleep disorders, respectively. Excess rates for narcolepsy were found for males (IRR:1.2, 95% CI, 1.0-1.5) and females (IRR:3.3, 95% CI, 3.0-4.1), and for sleep apnea in males (IRR:1.8, 95% CI, 1.5-2.2). Males and females had IRRs of 4.1 (95% CI, 3.1-5.5) and 7.0 (95% CI, 4.8-10.1), respectively, 6 months after being diagnosed with a sleep disorder.

**Conclusions:**

Sleep disorders were associated with higher suicide rates even after adjusting for pre-existing mental disorders. Our findings suggest attention towards suicidal ideation in patients suffering from sleep disorders is warranted.

**Disclosure:**

Disclosures and Acknowledgements: Adam Spira has received honoraria for serving as a consultant to Merck and from Springer Nature Switzerland AG for guest editing special issues of Current Sleep Medicine Reports. The other authors report no conflict of in

